# *TMEM105* upregulation promotes colorectal cancer malignancy: a novel prognostic biomarker potentially linked to the MYC-Ribosome biogenesis axis

**DOI:** 10.1186/s12935-025-04156-4

**Published:** 2026-01-09

**Authors:** Ahmad Rezaenasab, Seyed Jalal Zargar, Maryam Peymani, Kamran Ghaedi

**Affiliations:** 1 Department of Cell & Molecular Biology, School of Biology, College of Science, University of Tehran, P.O. Box: 14155-6455, Tehran, Iran; 2 Department of Biology, ShK.C., Islamic Azad University, Shahrekord, Iran; 3 Cell and Molecular Biology and Microbiology, University of Isfahan, Isfahan, Iran

## Abstract

Transmembrane protein 105 (*TMEM105* ) has recently emerged as a potential oncogenic factor in various malignancies, yet its role in colorectal cancer (CRC) remains unclear. In this study, we comprehensively investigated the expression and function of *TMEM105* in CRC through an integrated in silico, ex vivo, and in vitro approach. Publicly available datasets (TCGA, GSE41328, and GSE25070) were analyzed to assess *TMEM105* transcript expression and its association with clinicopathological features, followed by weighted gene co-expression network analysis to identify relevant biological pathways. The expression levels were further validated via RT‒qPCR in 25 paired CRC and adjacent non-tumorous tissues. Functional assays were performed after siRNA-mediated silencing of *TMEM105* in CRC cell lines to evaluate its impact on cell viability, clonogenicity, migration, apoptosis, and pathway-specific gene expression. *TMEM105* was significantly upregulated in CRC tissues, and elevated expression levels were correlated with advanced stage (stage IV) and metastasis. Co-expression analysis revealed ribosome biogenesis and MYC signaling as pathways strongly associated with *TMEM105*. The functional inhibition of *TMEM105* reduced cell viability, impaired colony formation, suppressed migration, and promoted apoptosis, accompanied by the downregulation of the ribosomal genes *RPL7* and *RPS2* and a marked decrease in total protein synthesis. Collectively, these findings establish *TMEM105* as a putative oncogenic driver in CRC. Our data reveal a strong correlation between *TMEM105* upregulation and the modulation of ribosome biogenesis, a process known to be driven by MYC signaling, which aligns with recent mechanistic evidence in other malignancies.

**Supplementary Information:**

The online version contains supplementary material available at 10.1186/s12935-025-04156-4.

## Introduction

Colorectal cancer (CRC) is the third most commonly diagnosed cancer and the second leading cause of cancer-related deaths globally [1]. Its development is a multistep process characterized by the accumulation of genetic and epigenetic alterations, including mutations in tumor suppressors and oncogenes such as APC, MLH1, and KRAS, along with lifestyle-associated risk factors such as red meat consumption, obesity, and smoking [2, 3]. Inherited syndromes such as Lynch syndrome and familial adenomatous polyposis (FAP) further increase the risk of CRC [4]. Among the molecular changes contributing to CRC progression, dysregulation of gene expression plays a critical role in modulating tumor progression, immune evasion, and the response to therapy, highlighting its relevance in early detection and targeted treatment strategies [5, 6].

Dysregulation of gene expression, involving complex networks of transcripts, plays a critical role in modulating tumor progression. Recently, regulatory RNAs and transcripts with non-coding potential have garnered considerable attention as key drivers of cancer biology. Owing to their unique structures, these molecules engage in intricate interactions with DNA, RNA, and proteins, modulating chromatin remodeling and transcriptional control [7, 8]. In CRC, aberrant expression of such transcripts has been implicated in biological processes including epithelial‒mesenchymal transition (EMT), apoptosis, proliferation, and metabolism [9]. Prominent examples include *MIR17HG* and *FTX*, which promote malignancy through complex regulatory networks [10, 11].

Transmembrane protein 105 (*TMEM105*) is encoded by a gene located at chromosome 17q25.3 which, despite its protein-coding designation, has been increasingly characterized as a functional lncRNA. It is expressed in multiple tissues, including intestinal epithelial cells. While *TMEM105* has been associated with cancer progression in other tumor types, its role in CRC remains unexplored. In breast cancer, elevated *TMEM105* expression is correlated with poor prognosis and enhanced liver metastasis [12]. Mechanistically, *TMEM105* exhibits a hallmark lncRNA function by acting as a competitive endogenous RNA (ceRNA) to sponge miR-1208, thereby increasing LDHA expression and promoting glycolysis. This interaction creates a positive feedback loop via the SHH–MAZ signaling pathway that further amplifies *TMEM105* expression [12]. Similarly, in pancreatic ductal adenocarcinoma (PDAC), *TMEM105* has been identified as a disulfidptosis-related lncRNA that stabilizes β-catenin, enhances c-MYC and GLUT1 expression, and drives tumor proliferation and metabolic reprogramming [13].

Despite compelling evidence of the oncogenic potential of *TMEM105* in other malignancies, its expression profile and functional role in CRC have not been characterized. In this study, we aimed to investigate the expression dynamics and potential oncogenic role of *TMEM105* in CRC. We first conducted in silico analyses to evaluate *TMEM105* expression and its association with cancer-related signaling pathways via publicly available transcriptomic datasets. We subsequently validated these observations through comparative expression analysis of paired CRC tumor and adjacent non-tumorous tissues. Finally, we performed in vitro experiments to assess the impact of *TMEM105* silencing on CRC cell survival, migration, apoptosis, and clonogenic potential. Overall, this work sought to uncover the functional relevance of *TMEM105* in CRC carcinogenesis and evaluate its utility as a novel biomarker and therapeutic target.

## Materials and methods

### Study design and overview

**Fig. 1:**
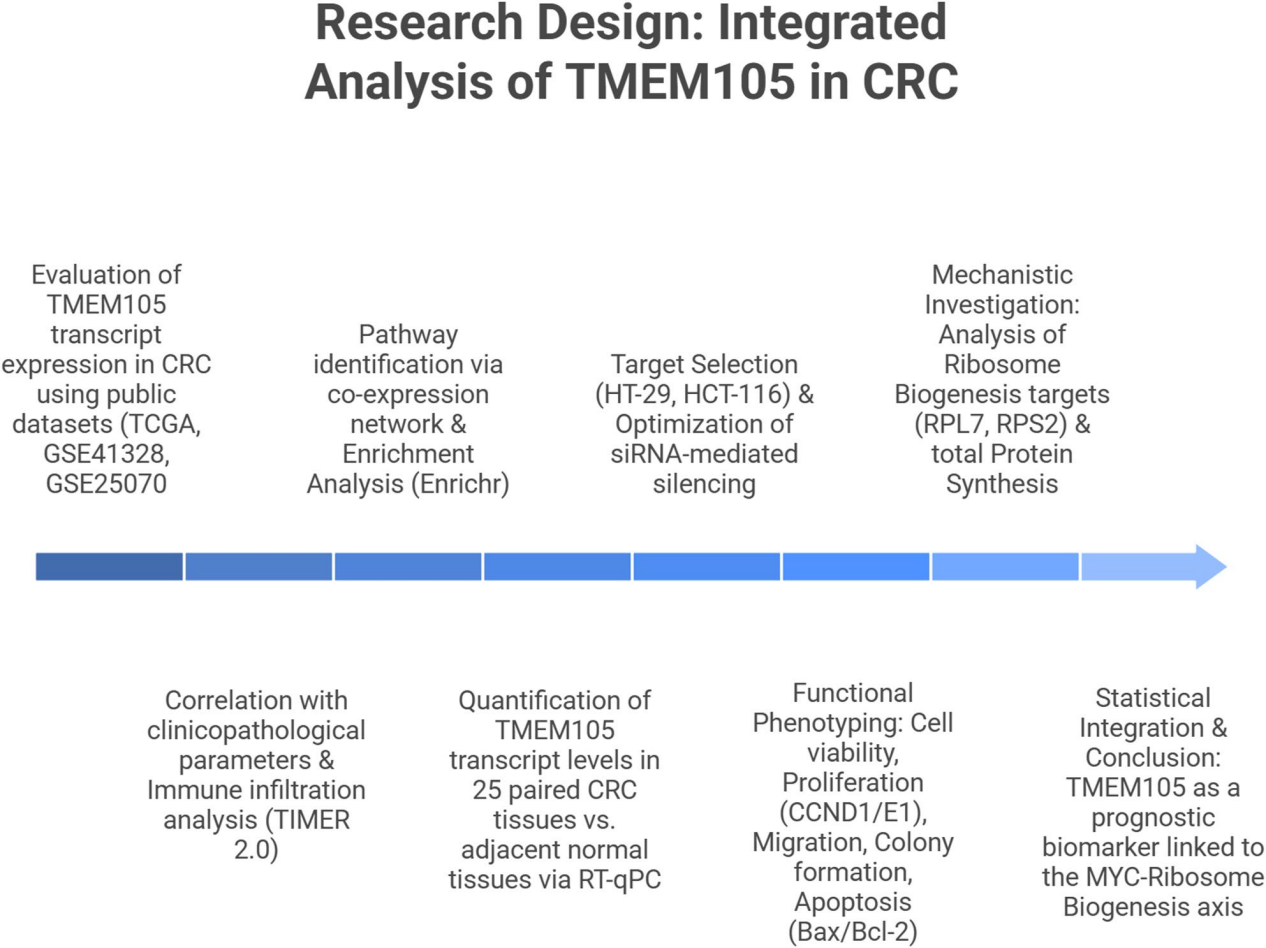
Schematic representation of the integrated research workflow investigating the role of *TMEM105* in cancer progression. The diagram illustrates a comprehensive multi-phase approach, beginning with the formulation of the research question and hypothesis, followed by in silico analyses, including differential expression profiling (TCGA and GEO datasets), correlation with clinicopathological parameters, and co-expression network with pathway enrichment analysis. Target genes identified computationally were validated ex vivo via RT-qPCR in patient-derived tissues, and in vitro functional assays were used to assess cell viability, proliferation, migration, colony formation, and apoptosis, alongside marker analysis (BAX, BCL-2, Cyclin E1, and Cyclin D1). Validation was performed using patient-derived tissues (ex vivo) and cell line models (in vitro) to evaluate tumor growth and metastatic potential. Mechanistic insights were explored through ribosome biogenesis, MYC signaling, and immune infiltration analyses. The expanded clinical data analysis incorporated survival analysis and ROC curve assessment. Data from all experimental tiers were statistically integrated to derive final conclusions, underscoring the potential of *TMEM105* as a prognostic biomarker and therapeutic target

This study was designed as a multiphase investigation aimed at elucidating the role of *TMEM105* in cancer progression by integrating computational, experimental, and clinical approaches. The methodological framework was structured to ensure both analytical rigor and translational relevance, enabling the systematic exploration of *TMEM105* expression patterns, functional roles, and mechanistic pathways across multiple experimental tiers. The research framework encompassed sequential in silico analyses, ex vivo validation using clinical samples, in vitro functional assays, and mechanistic exploration. The overall workflow of the study, which integrates these complementary approaches, is illustrated in Fig. 1, providing a stepwise overview from hypothesis formulation to clinical interpretation. This schematic serves as a visual guide to the logical progression of the research and the interconnection between its analytical and experimental components. The sample size for the clinical cohort was determined on the basis of precedent from similar studies and the availability of patient samples. For in vitro assays, the number of experimental replicates was selected to provide adequate statistical power to detect significant differences between groups.

### Data sources and preprocessing

To investigate the differential expression of *TMEM105* and its potential association with colorectal cancer (CRC) malignancy, RNA-sequencing data from The Cancer Genome Atlas (TCGA) were employed. Raw count data in STAR-counts format were retrieved via the TCGAbiolinks R package [14]. For TCGA RNA-seq data, the ‘voom’ function within the limma-voom package was utilized for data preprocessing [15]. The resulting expression matrix served as the basis for all subsequent analyses. The TCGA cohort comprised 483 tumor samples and 41 non-tumor tissues spanning multiple clinical stages. Updated clinical metadata were incorporated to evaluate the relationships between *TMEM105* expression and relevant clinical parameters. In addition, two independent GEO datasets (GSE41328 and GSE25070), consisting of paired tumor and adjacent normal tissues, were analyzed. The raw microarray data were processed using the limma package, which included background correction, RMA normalization, and log2 transformation [15].

### Co-expression network and clustering analysis

To identify genes co-expressed with *TMEM105*, Pearson correlation coefficients were computed between *TMEM105* and all other genes in the normalized TCGA expression matrix. Genes with correlation coefficients > 0.5 and p-values < 0.01 were retained for downstream analysis, including pathway enrichment. The mentioned criterion serves as an empirical measure that can identify genes with a putative functional association with *TMEM105*. Co-expression networks were visualized in Cytoscape. CRC tumor samples were then clustered based on expression profiles of *TMEM105*-associated genes. The optimal number of clusters was determined using the within-cluster sum of squares (WSS) method. K-means clustering was subsequently applied to divide the samples into two clusters: Cluster C1 (low *TMEM105* expression) and Cluster C2 (high *TMEM105* expression). The algorithm process was repeated 100 times with 1,000 centroid repositionings to ensure robust classification.

### Differential expression and pathway enrichment analysis

In the TCGA dataset, samples were grouped into tumor and non-tumor tissue categories based on clinical annotations, and paired tumor/normal tissues were applied to the GSE41328 and GSE25070 datasets. Differential gene expression analysis was performed using a linear modeling approach via the limma package [15]. A false discovery rate (FDR) of less than 0.01 was used to determine statistical significance. Differentially expressed genes between Clusters C2 and C1 were also identified to facilitate pathway analysis. Enrichment analysis of cluster-specific differentially expressed genes (DEGs) and the *TMEM105* co-expression network was performed via the Enrichr web-based platform (https://maayanlab.cloud/Enrichr/). Annotations were retrieved from the KEGG and MSigDB (Hallmark gene sets) databases. To identify the most biologically relevant pathways, terms were ranked based on statistical significance (from lowest to highest FDR). An FDR < 0.01 was considered statistically significant.

### Immune cell infiltration analysis

To evaluate the relationship between *TMEM105* expression and immune cell infiltration in CRC, the TIMER 2.0 web server (https://timer.cistrome.org/) was used. Outputs from the EPIC and TIMER algorithms were considered. Only statistically significant associations between *TMEM105* expression and immune cell populations have been reported.

### Tissue collection and ex vivo samples

A total of 25 CRC tumors and matched adjacent healthy tissues were collected from the Iran Tumor Bank at Imam Khomeini Hospital, Tehran. The diagnosis of cancer and healthy tissue was performed by a certified pathologist. The samples were immediately frozen in liquid nitrogen and stored until testing. All patients provided written informed consent. The study protocol was approved by the Research Ethics Committee of the University of Tehran in compliance with guidelines from the Iranian Ministry of Health. The study cohort included 25 patients with a histologically confirmed diagnosis of colorectal cancer, comprising 15 males and 10 females. Regarding age, 19 patients were over 50 years old, and 6 were under 50 years old. All collected samples met the predefined inclusion criteria and were used for subsequent analysis, with no sample attrition during the study. The clinical characteristics of the patients are detailed in Table 1.

**Table 1:** Clinical information for CRC samples

characteristic	Number (*N* = 25)
Age < 50 > 50	6 19
Gender Male Female	15 10
TNM stage I II III IV	3 9 8 5
Tumor size < 5 cm > 5 cm	14 11

### Cell culture

HT-29 and HCT-116 human CRC cell lines were purchased from the Pasteur Institute of Iran. The cells were cultured in DMEM supplemented with 10% fetal bovine serum (FBS), 1% penicillin‒streptomycin (100 U/mL penicillin and 100 µg/mL streptomycin), and GlutaMAX™. Cultures were maintained at 37 °C in a humidified 5% CO₂ incubator. The cell lines were obtained from the Pasteur Institute of Iran, where their authenticity was certified by the supplier, and they were confirmed to be free of mycoplasma contamination. The cells were subcultured at 80–90% confluence with 0.25% trypsin-EDTA and cryopreserved with a mixture of 90% FBS and 10% DMSO.

### SiRNA design and cell transfection

Target-specific siRNAs for *TMEM105* were designed via the siDirect online tool (https://sidirect2.rnai.jp/) and are listed in Table 2. All primers and siRNA sequences were explicitly designed to target the NCBI RefSeq transcript of *TMEM105* (Accession: NR_165247.1) to ensure specificity. Transfections of HT-29 and HCT-116 cells were carried out via Lipofectamine™ (Invitrogen, USA). Initial optimization of the transfection conditions was performed by varying the Lipofectamine volume (0.2–2 µL/100 µL culture medium) and siRNA concentration (5–80 nM), with cytotoxicity assessed via the MTT assay.

**Table 2:** The sequence list and information of the primers and primers used in this study are summarized in the table below

Gene Names	Forward primer (5’->3’)	Reverse primer (5’->3’)	Tm	NCBI accession number
CCND1	GCTCACGCTTACCTCAACCA	ATCCAGGACTTGTGCCCTTG	60	NM_053056.3
CCNE1	ATACTTGCTGCTTCGGCCTT	TCAGTTTTGAGCTCCCCGTC	58	NM_001238.4 NM_001322259.2 NM_001322261.2 NM_001322262.2 NM_001440305.1 NM_001440306.1 NM_001440307.1
BCL2	GAACTGGGGGAGGATTGTGG	GCCGGTTCAGGTACTCAGTC	60	NM_000633.3 NM_000657.3 NM_001438935.1
BAX	CCCCGAGAGGTCTTTTTCCG	GCACAGGGCCTTGAGCAC	60	NM_001291428.2 NM_001291429.2 NM_001291430.2 NM_001291431.2 NM_004324.4 NM_138761.4 NM_138763.4 NM_138764.5
GAPDH	GGGAGCCAAAAGGGTCATCA	GTGCTAAGCAGTTGGTGGTG	60	NM_001101.5
TMEM105	TCTCATCTCCCCACAGGAATC	TTTGCTTCTTAGCCCCCAACC		NR_165247.1
siRNA (scramble)	UUCUCCGAACGUGUCACGU	ACGUGACACGUUCGGAGAA	-	-
siRNA-1	CCCAUAGCUGACACUUCUA	UAGAAGUGUCAGCUAUGGG	-	NR_165247.1
siRNA-2	GGCAAGCUCUGAUCUUACA	UGUAAGAUCAGAGCUUGCC	-	NR_165247.1

The cells were seeded according to the manufacturer’s protocol such that they reached 60–70% confluence within 24 h at the time of transfection. To minimize potential bias from positional effects, the assignment of wells to the various treatment groups (e.g., Control, Scramble, siRNA-1, and siRNA-2) was randomized for each independent experiment. siRNAs and Lipofectamine were separately diluted in serum- and antibiotic-free media, incubated at room temperature for 5 min, combined, and incubated for an additional 15–20 min to form siRNA‒lipid complexes. The complexes were added to the cells and incubated for 8 h, after which the medium was replaced with complete culture medium (culture medium containing 10% FBS and antibiotics). The knockdown efficiency was measured via qRT‒PCR 24 to 48 h after transfection.

### MTT assay for cell viability

Cells were seeded in 96-well plates and allowed to adhere for 24 h, followed by siRNA treatment for 48 h. Then, 10 µL of MTT reagent was added to each well and incubated for 2 h. After the formazan crystals formed, they were solubilized with 100 µL of DMSO, and the absorbance was measured at 570 nm via an ELISA plate reader. Cell viability was calculated relative to that of the untreated controls.

### Wound healing assay (Cell Migration)

Migration was evaluated via a wound healing assay. HT-29 and HCT-116 cells were cultured to 70–80% confluence in 6-well plates. A uniform scratch was made using a 100-µL pipette tip, and the cells were washed with culture medium to remove debris. The cells were incubated in low-serum medium (1% FBS) and treated with siRNA. Initial scratch images (0 h) were captured, and the plates were incubated at 37 °C and 5% CO₂. Migration was assessed by imaging the same fields after 24 and 48 h. Wound closure was quantified via ImageJ, and migration percentages were calculated.

### Colony formation assay

HT-29 and HCT-116 cells were seeded in 6-well plates at 500 cells/well and treated with siRNA for 24 h. Colonies were allowed to form over 14 days, and the media was changed every 48 h. At the end of the incubation period, the medium was removed, and the cells were washed with PBS, fixed with methanol for 20 min, and stained with 0.5% crystal violet for 15 min. The plates were washed and air-dried, and the colonies were counted via ImageJ software.

### Annexin V/PI apoptosis assay

Apoptosis analysis was performed with an Annexin V-FITC/PI apoptosis detection kit according to the manufacturer’s instructions. Following 48 h of siRNA treatment, HT-29 and HCT-116 cells were harvested, washed with PBS, and detached with trypsin without EDTA. The cells were subsequently centrifuged at 1,000 rpm for 5 min, after which the pellet was resuspended in 100 µL of binding buffer. Then, 5 µL of Annexin V-FITC and 5 µL of PI were added to each sample and incubated in the dark at room temperature for 15 min. Afterward, 400 µL of binding buffer was added, and the samples were analyzed by flow cytometry (FACSCalibur, USA). The fluorescence from Annexin V and PI was detected via the FL1 and FL3 channels, respectively. The data were analyzed via quadrant gating to quantify the percentages of viable (Annexin V⁻/PI⁻), early apoptotic (Annexin V⁺/PI⁻), late apoptotic (Annexin V⁺/PI⁺), and necrotic (Annexin V⁻/PI⁺) cells.

### RNA Extraction, cDNA Synthesis, and RT‒qPCR

Total RNA was extracted via TRIzol reagent following the manufacturer’s protocol. RNA purity and concentration were assessed via optical density at 260/280 nm, and equal amounts of RNA were used for cDNA synthesis. The samples were treated with DNase I to eliminate genomic DNA contamination. cDNA synthesis was performed via a commercial kit (Yekta Tajhiz), oligo (dT), random hexamer primers, and reverse transcriptase enzyme. Specific primers targeting the *TMEM105* transcript (NR_165247.1) were designed via Primer-BLAST and Oligo7 software; all the gene sequences are summarized in Table 2. Gene expression was quantified by RT‒qPCR using SYBR Green dye and gene-specific primers. GAPDH was used as the internal control. The expression levels were calculated via the 2⁻ΔCt method.

### Protein quantification via a BCA assay

Total protein concentration was determined via a BCA protein assay kit (Pars Tous, Iran) following the manufacturer’s protocol. A standard curve was generated via the use of serial dilutions of bovine serum albumin (BSA) in the appropriate lysis buffer, ranging from 0 to 2000 µg/mL. After treatment, equal numbers of cells were seeded for each group to reduce variability. The cells were washed three times with PBS to remove extracellular proteins, harvested, and lysed. The lysates were diluted to ensure that they fell within the linear range of the standard curve. In a 96-well plate, 25 µL of each sample or standard was added per well, followed by the addition of 200 µL of working reagent. After gentle mixing, the plates were incubated at 37 °C for 30 min, and the absorbance was measured at 562 nm via a microplate reader.

### Statistics and software

Preprocessing and analysis of TCGA and GEO data were conducted via the R programming language (v. 4.3.2). Statistical differences between paired tumor and normal tissues were assessed using the Paired t-test. For independent groups (e.g., cell line assays), the Student’s t-test or One-way ANOVA followed by Tukey’s post-hoc test was used. The false discovery rate (FDR) was employed to correct for multiple testing in high-throughput analyses. The Pearson correlation test was applied for the co-expression network analysis. To prevent observer bias during data acquisition and analysis, key quantitative steps, such as the analysis of wound healing assays, colony counting, and flow cytometry data, were performed by an investigator blinded to the treatment conditions. GraphPad Prism (v. 8.4) was used to generate graphical representations. A p-value < 0.05 was considered statistically significant.

## Results

### *TMEM105* transcript is upregulated in CRC and is correlated with clinical features

**Fig. 2:**
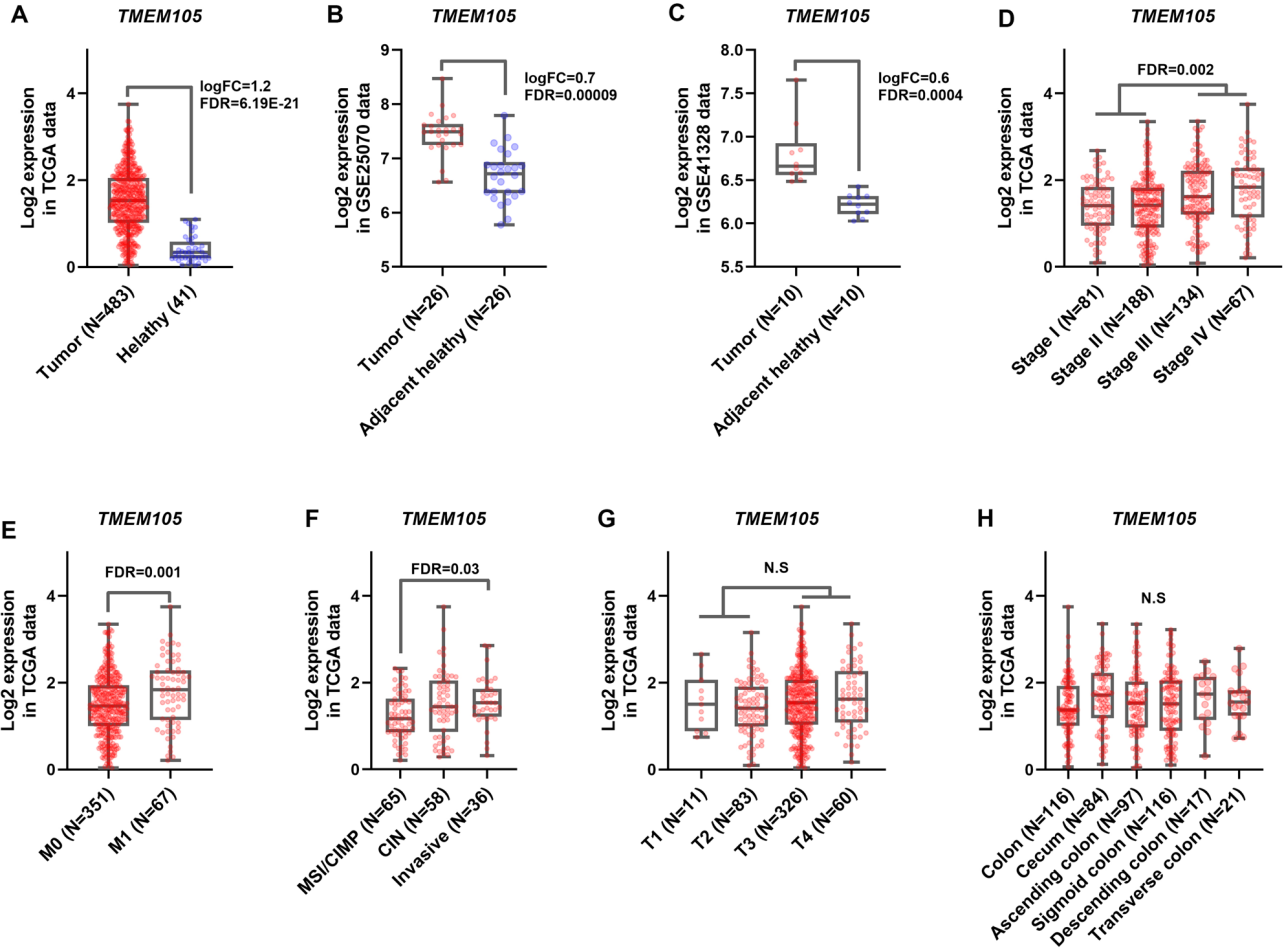
Upregulation of *TMEM105* relative RNA expression in colorectal cancer (CRC). (**A–C**) Comparative analysis of *TMEM105* transcript levels between tumor and normal tissues in the TCGA, GSE41328, and GSE25070 datasets. The RNA expression values were normalized and log2-transformed for visualization. (**D–H**) Correlations between *TMEM105* RNA expression levels and clinicopathological features were examined via TCGA data. *TMEM105* RNA expression was significantly associated with tumor stage, TNM-M classification, and distinct CRC subgroups. All graphs were based on transcriptomic data (logFC log fold change; FDR: false discovery rate)

In silico analysis of TCGA RNA-sequencing data revealed significant upregulation of *TMEM105* RNA expression in CRC tumor samples relative to healthy tissues, with a > 2-fold increase in transcript levels (Fig. 2A; logFC = 1.2, FDR < 0.0001). Consistently, paired analysis of tumor and adjacent non-tumorous samples (GSE41328 and GSE25070) revealed significantly elevated *TMEM105* RNA expression in tumor tissues (Fig. 2B and C; FDR < 0.0001).

Analysis of TCGA clinical annotations revealed that *TMEM105* transcript abundance was significantly higher in advanced-stage (III–IV) colorectal tumors than in early-stage (I–II) tumors (Fig. 2D; FDR < 0.01). Patients with metastatic disease (M1) also presented significantly elevated *TMEM105* RNA levels compared with nonmetastatic patients (M0) (Fig. 2E; FDR < 0.01). In addition, the invasive molecular subtype presented higher *TMEM105* transcript expression than the other subgroups did (Fig. 2F; FDR < 0.05). In contrast, *TMEM105* RNA expression did not differ significantly with respect to tumor anatomical location and TNM-M stage (Figs. 1H and 2G). These findings indicate that *TMEM105* is markedly upregulated at the transcriptional level in CRC and may be associated with features of tumor aggressiveness and malignancy-related features.

### *TMEM105* expression is correlated with genes involved in ribosome biogenesis and MYC targets

**Fig. 3:**
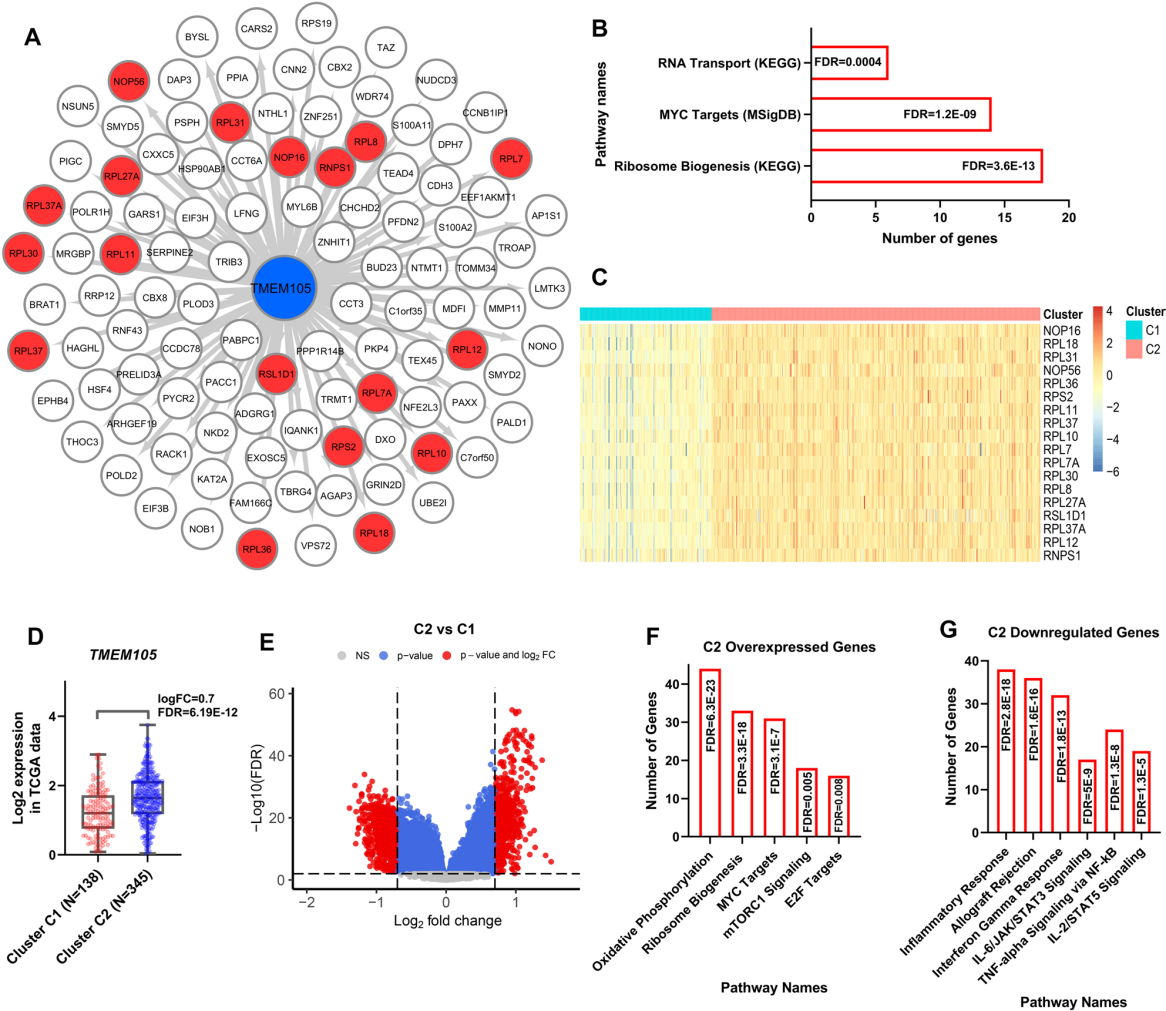
Co-expression analysis reveals that *TMEM105* upregulation is associated with ribosome biogenesis and MYC-driven pathways in colorectal cancer (CRC). (**A**) Co-expression network of genes significantly correlated with *TMEM105* expression in the TCGA dataset (Selection criteria Pearson correlation *r* > 0.5, *P* < 0.01). Red nodes indicate genes functionally annotated to the ribosome biogenesis pathway. (**B**) Bar chart displaying the top-ranking enriched pathways (sorted by lowest to highest FDR) for the *TMEM105* co-expression network. (**C**) K-means clustering of TCGA tumor samples based on the expression profiles of the 18 ribosome biogenesis–related genes (highlighted as red nodes in Panel A). This stratification classified samples into two distinct groups: C1 (low expression of candidate genes) and C2 (high expression of candidate genes). (**D**) *TMEM105* expression across the defined clusters, with significantly higher expression in cluster C2. (**E**) Volcano plot displaying genes that were differentially expressed between clusters C2 and C1. (**F–G**) Bar charts displaying the top-ranking enriched pathways (sorted by lowest to highest FDR) for genes upregulated (**F**) and downregulated (**G**) in the high-*TMEM105* expression cluster (C2) compared with the low expression cluster (C1). (logFC: log fold change; FDR: false discovery rate)

Co-expression network analysis of TCGA data identified 106 genes that were positively co-expressed with *TMEM105* (Pearson *r* > 0.5, *P* < 0.01) (Fig. 3A). Functional enrichment analysis via Enrichr revealed that these genes were predominantly associated with Ribosome Biogenesis and MYC Targets pathways, which emerged as the top-ranking pathways (ranked 1 st and 2nd, respectively; Fig. 3B, FDR < 0.01), highlighting a potential regulatory link. Given that MYC is a master regulator of ribosome biogenesis, these findings suggest a functional interplay between TMEM105 upregulation and MYC-driven oncogenic programs (Fig. 3B; FDR < 0.01).

To further dissect the clinical relevance of this association, we stratified tumor samples based specifically on the expression profiles of the 18 genes within this network that are functionally annotated to ribosome biogenesis (highlighted as red nodes in Fig. 3A). Using this functionally filtered 18-gene set, K-means clustering classified the samples into two distinct groups: Cluster C1 (*n* = 138), characterized by low expression, and Cluster C2 (*n* = 345), characterized by high expression (Fig. 3C). In support of the validity of these clusters, *TMEM105* expression was significantly elevated in Cluster C2 compared with C1 (Fig. 3D; log2FC = 0.7, FDR < 0.001), further supporting the biological coherence of the stratification. Differential expression analysis between these clusters revealed 639 upregulated genes (logFC > 0.7, FDR < 0.01) and 615 downregulated genes (logFC < −0.7, FDR < 0.01) in Cluster C2 (Fig. 3E). The functional enrichment of the genes upregulated in C2 was significantly associated with oxidative phosphorylation, ribosome biogenesis, MYC target pathways, mTORC1 signaling, and E2F target pathways (Fig. 3F). Notably, these pathways represented the top-ranking enriched terms based on statistical significance (FDR < 0.01). In contrast, the genes downregulated in C2 were predominantly associated with immune-related pathways (Fig. 3G; FDR < 0.01). These findings suggest that *TMEM105* upregulation is closely linked to activated ribosomal biogenesis and MYC-driven oncogenic programs in CRC pathogenesis.

### High *TMEM105* expression is associated with reduced immune cell infiltration

**Fig. 4:**
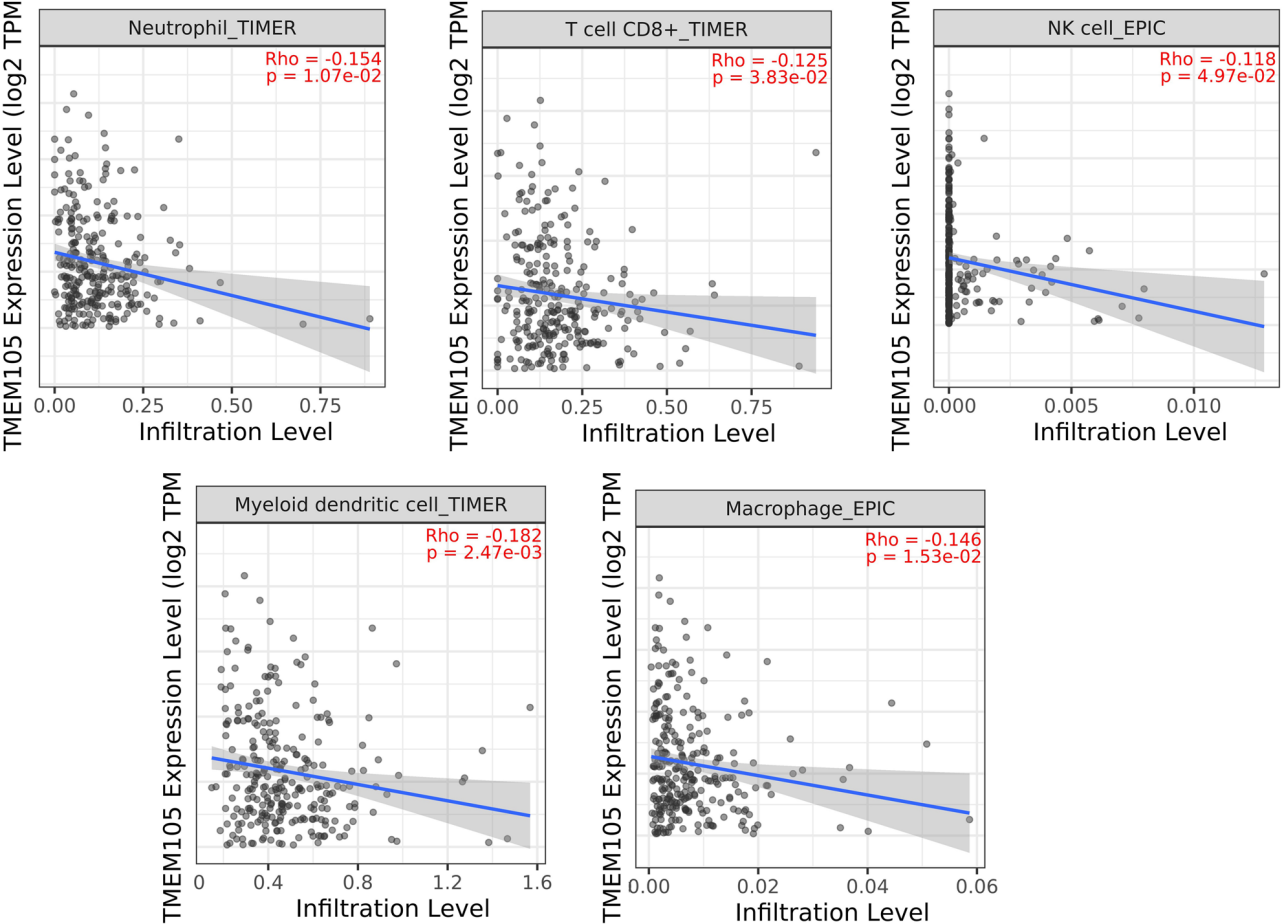
Association between *TMEM105* expression and immune cell infiltration in colorectal cancer (CRC). Immune cell infiltration levels were analyzed in relation to *TMEM105* expression via the TIMER 2.0 platform on the basis of TCGA-COAD data. (Rho Spearman’s correlation coefficient; p: p-value)

The relationship between *TMEM105* expression and immune cell infiltration in CRC was evaluated via the TIMER 2.0 web server. The immune cell composition was estimated in the TCGA dataset by employing the TIMER and EPIC computational deconvolution algorithms. A comprehensive analysis was performed across the full panel of immune cell types available in the database. Our results revealed that *TMEM105* expression exhibits a specific and statistically significant inverse correlation exclusively with CD8^+^ T cells, neutrophils, natural killer (NK) cells, dendritic cells (DCs), and macrophages (Fig. 4; *P* < 0.01). Analyses of other immune cell populations did not yield statistically significant associations (*P* > 0.05, data not shown), highlighting a selective immunomodulatory role for *TMEM105* in the tumor microenvironment. These results suggest that the overexpression of *TMEM105* may contribute to immune evasion in CRC by suppressing the recruitment of these immune cells to the TME. However, as these associations are based on in silico analysis, further experimental validation is needed to confirm the role of *TMEM105* in tumor-immune interactions.

### *TMEM105* is upregulated in tumor tissues, and its Silencing reduces the viability of CRC cell lines

**Fig. 5:**
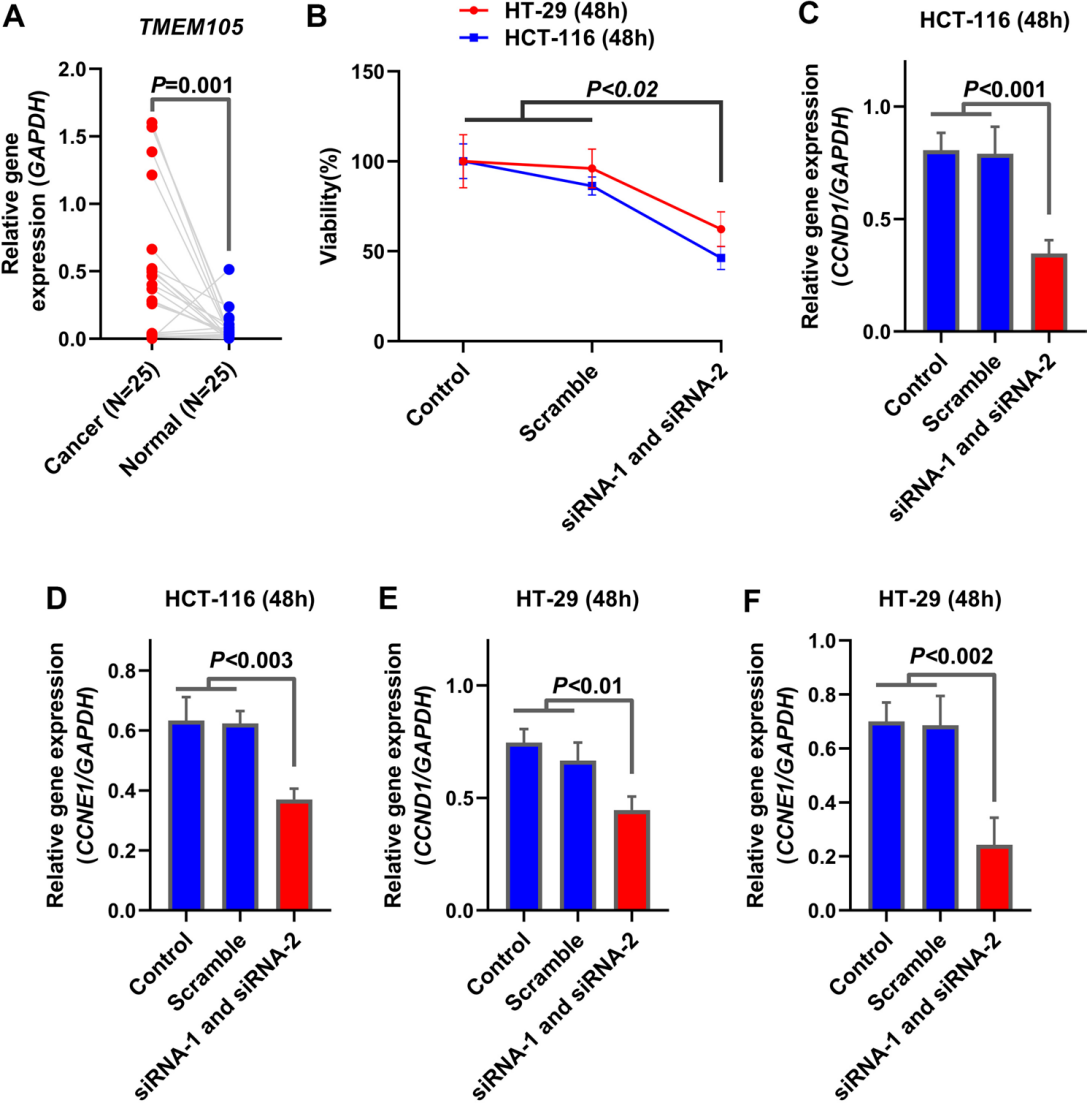
Upregulation of *TMEM105* in CRC tissues and the effect of its silencing on cell viability and proliferation. (**A**) Relative *TMEM105* mRNA expression in patient-derived CRC tissues compared with matched adjacent normal tissues (*n* = 25 per group), quantified via RT-qPCR (*P* = 0.001). (**B**) Cell viability of HT-29 and HCT-116 cells at 48 h post-transfection with control, scramble, or *TMEM105*-targeting siRNAs, determined by MTT assay. (**C–F**) Relative mRNA expression of cell cycle regulators CCND1 and CCNE1 in HCT-116 (**C**,** D**) and HT-29 (**E**,** F**) cells following *TMEM105* knockdown. Error bars represent standard deviation (SD). (p-values as indicated)

To validate our in silico findings, we quantified *TMEM105* expression in 25 paired CRC and adjacent healthy tissues via RT‒qPCR. Consistent with the bioinformatic data, *TMEM105* was significantly upregulated in tumor samples compared with adjacent non-tumorous tissues (Fig. 5A; *P* = 0.001).

For functional characterization, we selected HT-29 and HCT-116 cell lines based on their endogenous expression levels (Supplementary Figure S1A). We established optimal transfection conditions by evaluating lipofectamine cytotoxicity and titrating siRNA concentrations (Supplementary Figure S1B–E). RT‒qPCR confirmed that co-transfection with two siRNAs (siRNA-1 and siRNA-2) resulted in robust knockdown efficiency (> 50%) in both cell lines (Supplementary Figure S1F–I); thus, these conditions were utilized for all subsequent functional assays.

Assessing the biological impact of this knockdown revealed that *TMEM105* silencing significantly reduced the viability of CRC cells compared with control groups (Fig. 5B; *P* < 0.02). Moreover, the expression of key proliferation markers, Cyclin E1 (CCNE1) and Cyclin D1 (CCND1), was significantly downregulated following *TMEM105* depletion (Fig. 5C–F). Collectively, these findings indicate that *TMEM105* is crucial for CRC cell growth and survival and support its role as a candidate oncogene and a potential therapeutic target in CRC.

### Silencing *TMEM105* impairs migration and colony formation and promotes the apoptosis of CRC cells

**Fig. 6:**
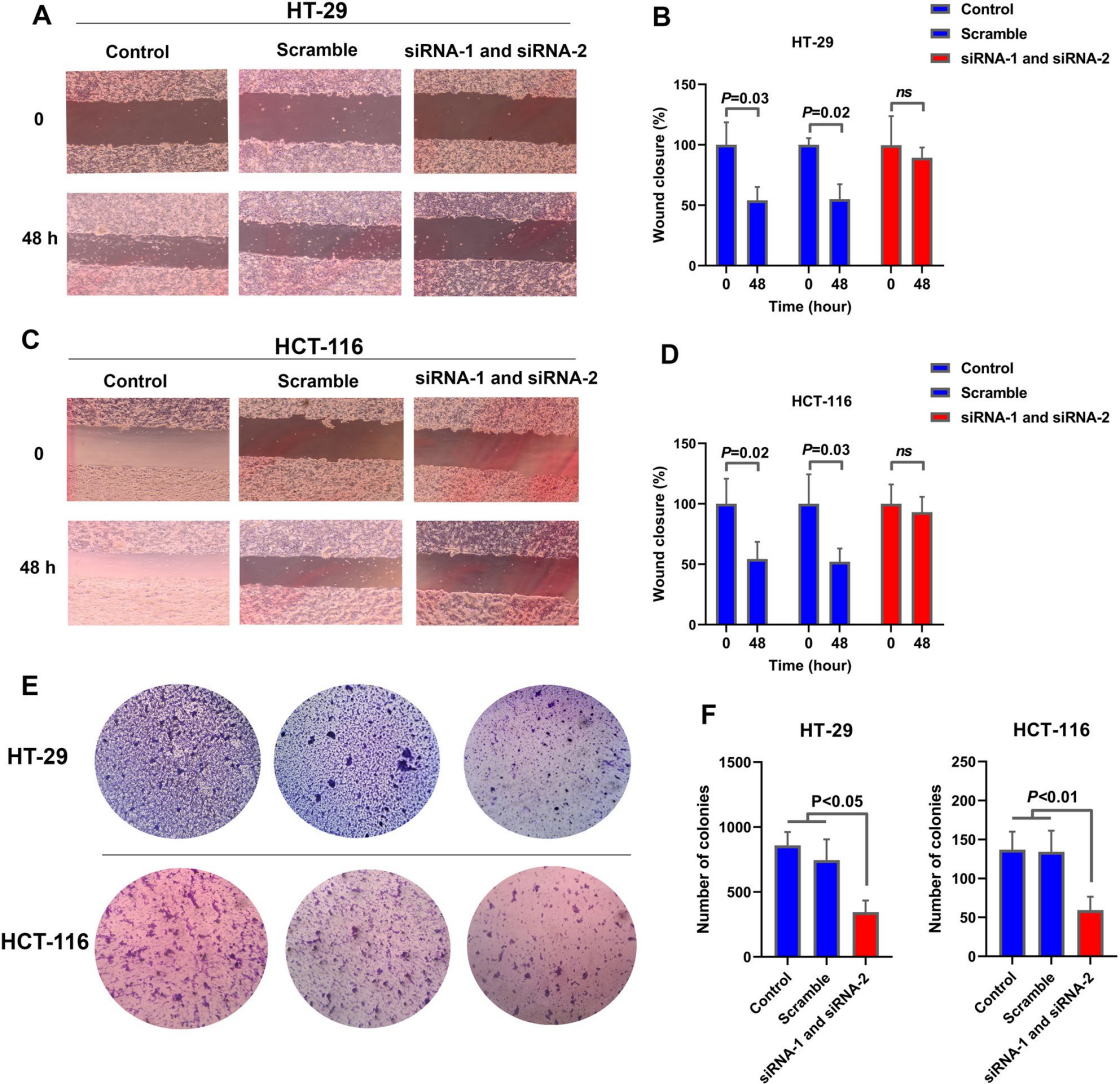
Impaired migration and colony formation following *TMEM105* knockdown. (**A–D**) Wound-healing assays in HT-29 and HCT-116 cells showing both qualitative and quantitative reductions in migratory capacity upon *TMEM105* silencing. (**E**,** F**) Colony formation assays comparing siRNA-treated cells with control cells revealed a significant decrease in colony number after *TMEM105* knockdown. The error bars represent the standard deviations

To comprehensively investigate the impact of *TMEM105* knockdown on the malignant phenotypes of CRC, we initially conducted wound healing assays to assess the migratory capacity of the cells. These assays demonstrated a significant decrease in the migration ability of both the HT-29 and HCT-116 cell lines following siRNA-mediated silencing of *TMEM105*. Quantitative analysis confirmed that this reduction was statistically significant, with *P* < 0.05 (Fig. 6A–D). These findings suggest that *TMEM105* plays a critical role in promoting CRC cell motility.

In the colony formation assays, compared with control cells, *TMEM105*-deficient cells presented a significantly lower clonogenic capacity (Fig. 6E–F; *P* < 0.05), suggesting impaired long-term proliferative potential.

**Fig. 7:**
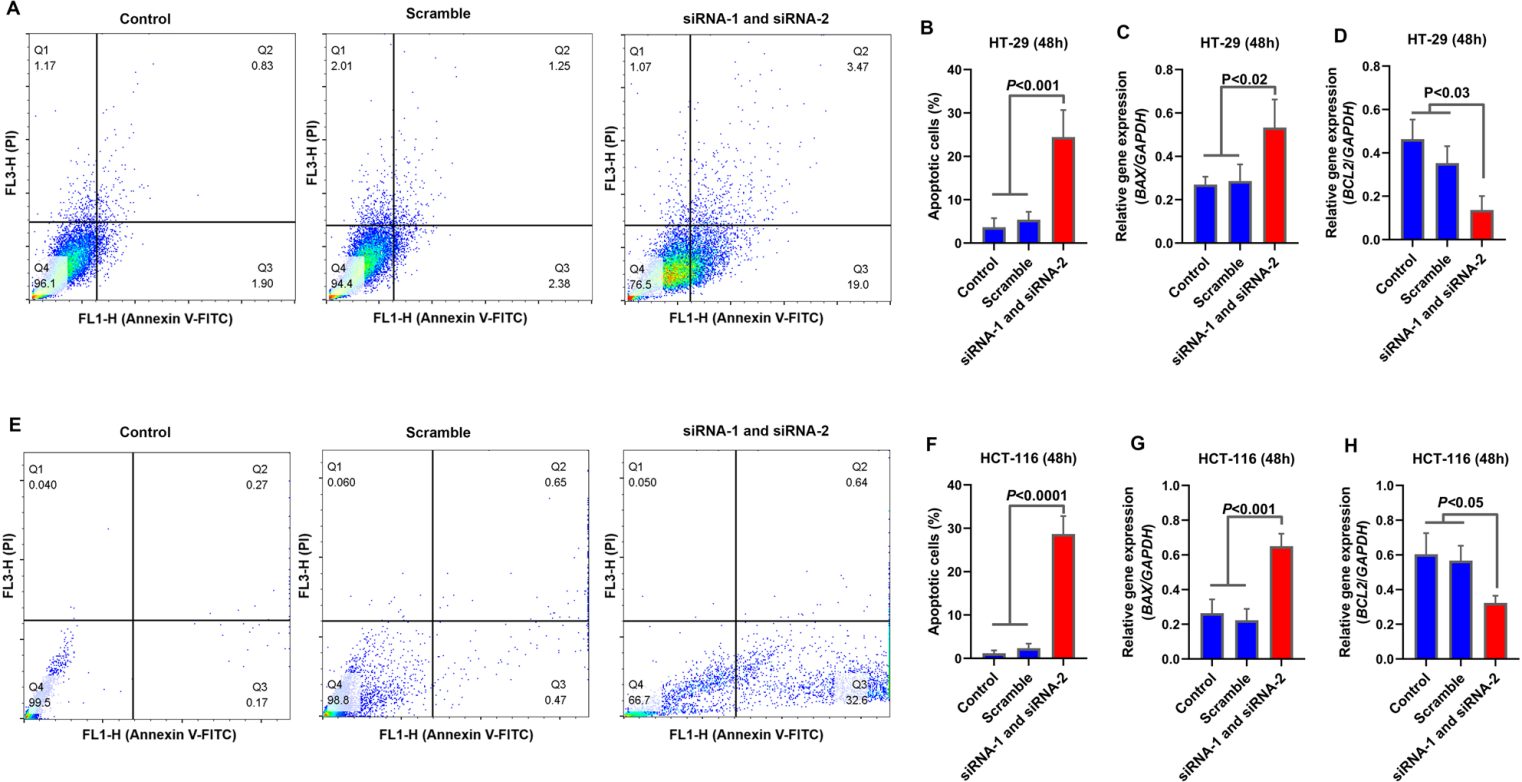
Enhanced apoptosis induced by *TMEM105* knockdown in CRC cells. (**A**,** B**) Apoptosis analysis of HT-29 cells after *TMEM105* silencing, which revealed a significant increase in the number of apoptotic cells. (**C**,** D**) Relative expression of apoptosis-associated markers (*BAX* and *BCL2*) in the siRNA-treated HT-29 cells compared with the control cells. (**E**,** F**) Annexin V/PI staining of HCT-116 cells, which revealed increased apoptosis following *TMEM105* knockdown. (**G**,** H**) Expression profiles of apoptosis-related genes (*BAX* and *BCL2*) in HCT 116 cells before and after *TMEM105* silencing

The apoptotic response following *TMEM105* knockdown was quantified via Annexin V-FITC/PI staining. Compared with those in the control and scrambled siRNA groups, the apoptotic population in the HT-29 cells increased by more than 20% (Fig. 7A–B; *P* < 0.001). This effect was accompanied by upregulation of the pro-apoptotic gene BAX and downregulation of the anti-apoptotic gene *BCL-2* (Fig. 7C–D; *P* < 0.05). Similar trends were observed in HCT-116 cells, where apoptosis increased by nearly 30% following *TMEM105* silencing, along with a shift in *BAX/BCL-2* expression, which was consistent with the activation of the intrinsic apoptotic pathway (Fig. 7E–H; *P* < 0.05).

These findings demonstrate that *TMEM105* promotes key oncogenic behaviors in CRC cells, including migration, clonogenicity, and survival, while its silencing induces apoptotic cell death, reinforcing its functional role in CRC tumor progression.

### *TMEM105* knockdown reduces ribosome Biogenesis-Related gene expression and total protein content

**Fig. 8:**
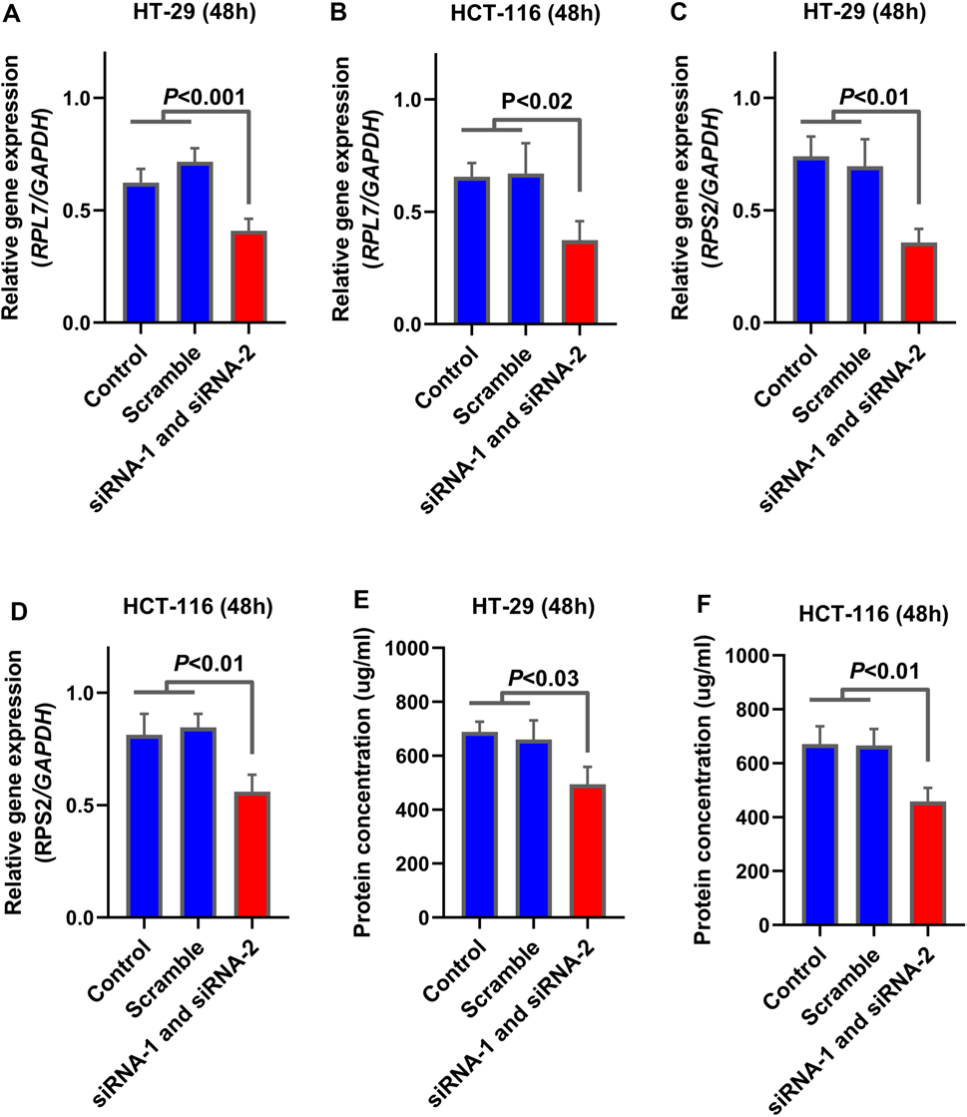
Knockdown of *TMEM105* downregulates the expression of ribosomal protein genes and attenuates total protein synthesis in colorectal cancer cells. (**A–D**) Relative mRNA expression of ribosomal protein genes (*RPL7* and *RPS2*) was quantified via RT‒qPCR in HT-29 and HCT-116 cells 48 h post-transfection with a non-targeting scramble siRNA or two independent siRNAs targeting *TMEM105* (siRNA-1 and siRNA-2). Significant downregulation of both genes was observed following *TMEM105* knockdown. (**E**,** F**) Consistent with these findings, quantification of the total cellular protein concentration revealed a significant reduction in both HT-29 (**E**) and HCT-116 (**F**) cells upon *TMEM105* silencing. The data are presented as the means ± SDs from three independent experiments. p-valus for the indicated comparisons are shown

Given the strong co-expression between *TMEM105* and ribosomal genes, the effect of *TMEM105* silencing on the representative ribosome biogenesis genes *RPS2* and *RPL7* was examined (Fig. 3, red nodes). RT‒qPCR analysis revealed significant downregulation of *RPS2* and *RPL7* in both HT-29 and HCT-116 cells following *TMEM105* knockdown (Fig. 8A and D; *P* < 0.05). Moreover, the total protein concentration was significantly reduced in *TMEM105*-depleted cells (Fig. 8E and F; *P* < 0.05), suggesting that *TMEM105* silencing correlates with reduced total protein synthesis. Collectively, these data suggest that *TMEM105* may promote CRC progression, potentially by supporting ribosome formation and total protein production—processes critical for tumor cell growth and metabolism.

## Discussion

Non-coding transcripts and regulatory RNAs are increasingly recognized as crucial modulators of cellular processes and have emerged as promising diagnostic biomarkers and therapeutic targets in a variety of diseases, including cancer [16]. With recent advances in RNA-based therapeutics—particularly antisense oligonucleotide drugs now progressing into phase II clinical trials—the landscape for targeting such regulatory transcripts is becoming increasingly viable and clinically relevant [17]. In the present study, we investigated the functional role of *TMEM105*, an understudied transcript, in the progression and malignancy of CRC.

Our integrated approach, which combines in silico analyses with ex vivo experiments on CRC tissues and cell lines, consistently revealed significant upregulation of *TMEM105* in tumor samples compared with adjacent healthy tissues. Notably, elevated *TMEM105* expression was significantly associated with advanced clinicopathological parameters, including stage III/IV tumors and metastatic status (TNM.M1). These observations are consistent with a growing body of evidence documenting the aberrant overexpression of *TMEM105* across a spectrum of human malignancies, thereby substantiating its role as a putative pan-cancer oncogenic factor. For example, in breast cancer, increased *TMEM105* expression has been linked to poor prognosis and increased metastatic potential [12]. Similarly, in pancreatic cancer [13], high *TMEM105* expression has been correlated with reduced overall survival [13]. Evidence also implicates *TMEM105* in the tumorigenesis of head and neck cancers, suggesting a broader oncogenic function [18]. In thyroid cancer, *TMEM105* appears to regulate cell cycle progression, with its overexpression correlating with unfavorable clinical outcomes [19]. Moreover, in gastric cancer, in silico data have demonstrated its upregulation and association with poor patient prognosis [20]. Collectively, these findings support a conserved oncogenic function of *TMEM105* across multiple cancer types and are consistent with its role in driving CRC aggressiveness, as observed in our study.

Functionally, our data demonstrated that silencing *TMEM105* significantly inhibited the viability, migration, and colony-forming capacity of CRC cell lines while simultaneously increasing their apoptotic activity. These results highlight *TMEM105* as a potential driver of tumor growth and cellular aggressiveness. Our findings are in line with earlier studies in breast cancer, which reported that suppressing *TMEM105* expression impairs cell migration and colony formation [12]. Additionally, research on pancreatic cancer has shown that depletion of *TMEM105* inhibits colony formation and migration, reinforcing its pro-tumorigenic role across malignancies [13].

Furthermore, to elucidate the molecular mechanisms underlying the protumorigenic functions of *TMEM105*, we performed our transcriptomic and functional enrichment analyses, which focused on pathways integral to ribosome biogenesis and protein synthesis. Specifically, co-expression network analysis revealed a strong positive correlation between *TMEM105* and several ribosome-related genes, including *RPS2* and *RPL7*, whose expression levels were markedly reduced following *TMEM105* knockdown. Notably, genes associated with MYC signaling pathways were also enriched among *TMEM105*-associated targets, suggesting that *TMEM105* may exert its oncogenic influence, at least in part, via the modulation of MYC-driven transcriptional programs. Given the central role of MYC in orchestrating ribosomal RNA transcription and ribosome assembly [21, 22], our findings provide mechanistic insight into how *TMEM105* may contribute to CRC pathogenesis through the enhancement of translational capacity and cellular proliferation.

While our study primarily relies on transcriptomic signatures to propose a link between *TMEM105* and the MYC-ribosome biogenesis axis, recent experimental evidence in other cancer models supports the plausibility of this regulatory connection at the protein level. Specifically, Yin et al. (2025) demonstrated in pancreatic cancer that *TMEM105* stabilizes β-catenin, which in turn enhances c-MYC protein expression [13]. This direct modulation of c-MYC by *TMEM105* provides a validated molecular scaffold for interpreting our findings in CRC. The robust co-expression of *TMEM105* with MYC targets and the significant reduction in ribosomal gene expression (*RPL7*, *RPS2*) and total protein synthesis observed in our study are consistent with this established axis. Thus, it is conceivable that *TMEM105* exerts a similar oncogenic function in CRC by sustaining MYC-driven transcriptional programs, although tissue-specific variations in this mechanism warrant further investigation.

Despite these compelling findings, several limitations should be acknowledged. First, our mechanistic insights into the MYC-ribosome biogenesis axis are primarily derived from co-expression analyses and downstream functional readouts (gene expression and total protein assays). While consistent with recent literature [13], direct protein-level validation of the *TMEM105*-MYC interaction (e.g., via Western blot or ChIP assays) in CRC tissues was not performed in this study. Second, the study relies on in vitro experiments using two CRC cell lines; future investigations utilizing in vivo models and comprehensive clinical cohorts are warranted to fully validate the translational potential of *TMEM105*-targeted interventions.

## Conclusion

Collectively, our findings establish *TMEM105* as a novel oncogenic transcript implicated in the progression of colorectal cancer (CRC). Its upregulation is significantly associated with adverse clinical features, whereas its silencing attenuates cardinal features of malignancy, including cell proliferation, migration, and survival. Mechanistically, our data reveal a compelling link between *TMEM105*, ribosome biogenesis, and MYC-driven transcriptional programs, providing a plausible axis for its oncogenic activity. Taken together, these results highlight *TMEM105* as a compelling candidate for further investigation, with potential as both a prognostic biomarker and a viable therapeutic target in CRC. Subsequent investigations utilizing in vivo models and comprehensive clinical cohorts are warranted to fully validate its clinical utility and explore the translational feasibility of *TMEM105*-targeted interventions.

## Supplementary Information

Supplementary Material 1.

## Data Availability

The datasets analyzed during the current study are available in the Cancer Genome Atlas (TCGA) (https://portal.gdc.cancer.gov/) and Gene Expression Omnibus (GEO) repositories under accession numbers GSE41328 and GSE25070. The original raw data generated during the current study are available from the corresponding author upon reasonable request.

## Abbreviations

TMEM105: Transmembrane protein 105
LncRNA: Long non coding RNA
CRC: Colorectal cancer
TCGA: The Cancer Genome Atlas
RT‒qPCR: reverse transcription quantitative polymerase chain reaction
SiRNA: Small interfering RNA
MYC: MYC proto-oncogene
TNM: tumor, node, metastasis (cancer staging system)
NK cells: Natural killer cells
DC cells: Dendritic cells
FDR: False discovery rate
LogFC: Log Fold Change
