# Supplementary material for: TMEM105 upregulation promotes colorectal cancer malignancy: a novel prognostic biomarker potentially linked to the MYC-Ribosome biogenesis axis
Source: Cancer Cell Int. 2026 Jan 9;26:76. doi: 10.1186/s12935-025-04156-4 (PMC12882376; doi:10.1186/s12935-025-04156-4)
Supplement: Supplementary file 1 — Supplementary Material 1. [file 12935_2025_4156_MOESM1_ESM.docx]

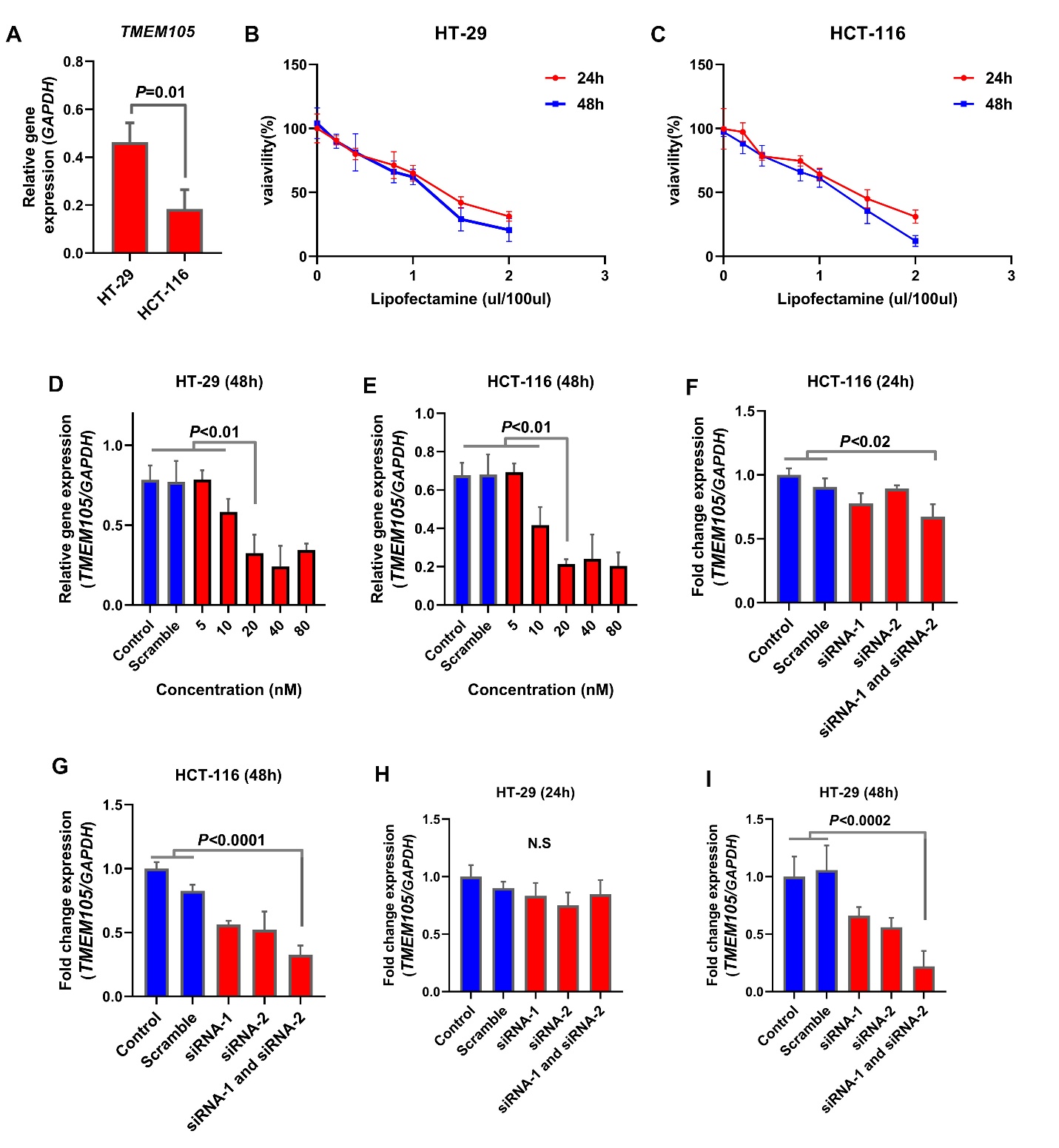


**Supplementary Figure S1. Optimization of siRNA transfection conditions and validation of *TMEM105* knockdown efficiency.** (A) Comparative analysis of endogenous *TMEM105* mRNA expression levels in HT-29 and HCT-116 colorectal cancer cell lines, revealing significantly higher baseline expression in HT-29 cells (P=0.01). (B, C) Evaluation of Lipofectamine-induced cytotoxicity in HT-29 (B) and HCT-116 (C) cells treated with varying reagent volumes (0–2 μL/100 μL). Cell viability was assessed over 24 h and 48 h to determine the optimal non-toxic concentration. (D, E) Optimization of siRNA concentration. Relative *TMEM105* expression in HT-29 (D) and HCT-116 (E) cells following treatment with a gradient of siRNA concentrations (5–80 nM). The 20 nM concentration was identified as effective for downstream assays. (F–I) Assessment of knockdown efficiency and time-dependent stability. Relative *TMEM105* expression was quantified in HCT-116 (F, G) and HT-29 (H, I) cells at 24 h and 48 h post-transfection with individual (siRNA-1, siRNA-2) or combined siRNAs. The combination of siRNA-1 and siRNA-2 yielded the most significant and sustained suppression compared with the scramble control. (P-values as indicated).
